# Predictive Significance of Tumour Size in Patients Undergoing Curative Surgery for Colorectal Cancer: A Retrospective Cohort Study

**DOI:** 10.7759/cureus.26656

**Published:** 2022-07-08

**Authors:** Shahab Hajibandeh, Mohammed Barghash, Rao Muhammad A Khan, David Milgrom, Saira Ali, Sofia Ali, Baqar Ali

**Affiliations:** 1 Department of General Surgery, Wrexham Maelor Hospital, Betsi Cadwaladr University Health Board, Wrexham, GBR; 2 Department of General Surgery, North Manchester General Hospital, Manchester University NHS Foundation Trust, Manchester, GBR; 3 Department of General and Colorectal Surgery, The Royal Oldham Hospital, Northern Care Alliance, Oldham, GBR; 4 Department of Vascular Surgery, Royal Preston Hospital, Lancashire Teaching Hospitals NHS Foundation Trust, Preston, GBR; 5 Bowel Cancer Screening, Harrogate and District NHS Foundation Trust, Harrogate, GBR

**Keywords:** risk prediction, rectal cancer, colonic cancer, prognosis, tumor size

## Abstract

Objectives

To evaluate the predictive significance of tumour size in patients undergoing curative surgery for colorectal cancer (CRC).

Methods

All patients undergoing curative surgery for colon or rectum cancer performed by a single colorectal surgeon between January 2013 and January 2020 were considered eligible for inclusion. Linear and binary logistic regression analyses were modelled to assess whether colonic or rectal tumour size could predict R0 resection, specimen length, number of harvested and positive lymph nodes, lymphocytic infiltration, venous invasion, and overall survival.

Results

A total of 192 patients were eligible for inclusion. In patients with colon cancer, tumour size was the independent predictor of the number of harvested lymph nodes (P<0.001), the number of positive lymph nodes (P=0.001), and lymphocytic infiltration (P=0.009). However, it did not predict R0 resection (P=0.563), specimen length (P=0.111), specimen length >120 mm (P=0.186), >12 harvested lymph nodes (P=0.145), venous invasion (P=0.103), and five-year overall survival (P=0.543). In patients with rectal cancer, tumour size was the independent predictor of the number of harvested lymph nodes (P<0.001) and the number of positive lymph nodes (P<0.001). However, it did not predict R0 resection (P=0.108), specimen length (P=0.774), specimen length >120 mm (P=0.405), >12 harvested lymph nodes (P= 0.069), lymphocytic infiltration (P=0.912), venous invasion (P= 0.105), and five-year overall survival (P=0.413).

Conclusions

The results of the current study suggest that tumour size on its own may not have a significant predictive value in oncological or survival outcomes in patients undergoing curative surgery for colon or rectum cancer.

## Introduction

The incidence of colorectal cancer (CRC) continues to increase steadily worldwide [1]. CRC is considered the fourth most commonly diagnosed cancer and the third most deadly cancer worldwide [1]. Despite the increasing incidence of CRC, the mortality associated with CRC has been reduced in developed countries due to the implementation of screening programmes and advances in management options, including newer surgical techniques and adjuvant and neoadjuvant chemotherapy regimens [1]. Consequently, there is an ongoing effort to identify new prognostic predictors in patients with CRC so that new treatment strategies can be developed to achieve better long-term outcomes.

The primary treatment modality for CRC is surgical resection. Then, histopathological analysis of the resected specimen is the most powerful method for assessing prognosis [2]. This prognostic assessment is crucial for surveillance purposes and also plays an essential part in the selection process for adjuvant therapy [3]. 

The prognostic significance of tumour size is reflected by its major role in the T-stage of many solid tumours, including breast, lung, renal and thyroid cancers. This is reflected in the first and seventh editions of the Cancer Staging Manual of the American Joint Commission on Cancer (AJCC), published in 1977 and 2012, respectively [4].

Tumour size calculated from the widest horizontal tumour diameter is a common medical parameter which has long been studied. However, despite the value of tumour size as a prognostic indicator in many other solid tumours, this has not been incorporated into Tumour (T), Nodal status (N), Metastasis (M) staging system for CRC [4].

The predictive significance of tumour size in patients with CRC has been controversial. While some studies concluded that tumour size has no prognostic significance in patients with CRC [5,6], tumour size has been identified as a predictor of oncological and survival outcomes in some studies [3,7,8]. Given the ongoing controversy, we aimed to evaluate the predictive significance of tumour size in patients undergoing curative surgery for CRC. Furthermore, considering that colon and rectal cancers have different disease characteristics and treatment options, we aimed to analyse the patients with colon and rectal cancers separately.

This article was previously presented as a meeting abstract at the Association of Great Britain & Ireland (ASGBI) - Future Surgery Virtual Congress on May 4, 2021 [9].

## Materials and methods

Study design and patient selection

We conducted a retrospective analysis of prospectively collected data on a cohort of patients operated by a single colorectal surgeon in a single centre located in the North West of England to evaluate the predictive significance of tumour size in patients undergoing curative surgery for CRC. Considering the retrospective nature of this study, patient consent and approval by Research Ethics Committees were not required. The study period was between January 2013 and January 2020. All consecutive adult patients undergoing surgery performed by a single colorectal surgeon with the curative intention for colon or rectum cancer were considered eligible for inclusion. The procedures of interest included laparoscopic, laparoscopic-assisted, or open right hemicolectomy, extended right hemicolectomy, segmental colectomy, left hemicolectomy, extended left hemicolectomy, anterior resection, and abdominoperineal resection. We excluded the patients who underwent an operation with palliative intention.

Outcome measures

The outcomes of this study included tumour size, R0 resection, specimen length, specimen length >120 mm, number of harvested lymph nodes, >12 harvested lymph nodes, number of positive lymph nodes, lymphocytic infiltration, venous invasion, and overall survival. Histological assessment of the resected specimen was used to determine tumour size (defined as the widest horizontal diameter of tumour) and the above specimen-related outcomes. R0 resection was defined as a microscopically margin-negative resection in which no gross or microscopic tumour remains in the primary tumour bed. Overall survival was defined as patients' survival in one, three and five years following their procedures.

Data collection

An electronic data collection proforma was created, which included data on the following parameters: patients' demographic data (age and sex), American Society of Anaesthesiologists (ASA) score, emergency or elective setting, surgical approach (laparoscopic or open), location of the tumour, performed procedure, tumour stage, tumour histology, and the aforementioned outcome measures.

Data synthesis and statistical analyses

Patients with colon cancer and patients with rectal cancer were analysed separately. The statistical analyses were performed using MedCalc 13.0 software (MedCalc Software, Ostend, Belgium). Simple descriptive statistics were used to present the baseline characteristics. Data were summarised with mean ± SD for continuous variables and frequencies or percentages for categorical variables. Linear and binary logistic regression analyses were modelled to assess whether colonic or rectal tumour size could predict R0 resection, specimen length, specimen length >120 mm, number of harvested lymph nodes, >12 harvested lymph nodes, number of positive lymph nodes, lymphocytic infiltration, venous invasion, and overall survival. Tumour size was considered an independent variable, and R0 resection, specimen length, specimen length >120 mm, number of harvested lymph nodes, >12 harvested lymph nodes, number of positive lymph nodes, lymphocytic infiltration, venous invasion, and overall survival, each was separately considered as dependent variables. When regression analyses identified tumour size as a predictor of a variable, receiver operating characteristic (ROC) curve analysis was performed using the method described by DeLong et al. to determine the cut-off value of tumour size that could predict the variable. We reported relevant area under the curve (AUC), sensitivity and specificity for the determined cut-off value. All statistical tests were two-tailed and statistical significance was assumed at P < 0.05.

## Results

**Figure 1:**
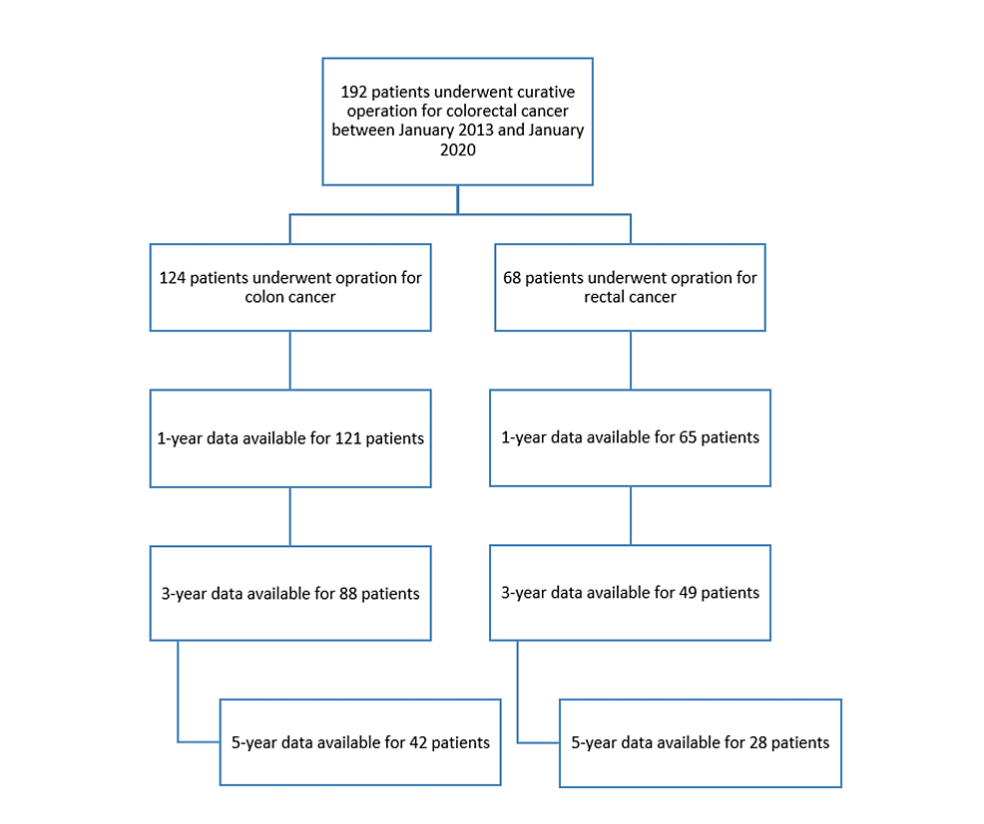
Study flow chart.

A total of 192 patients were considered eligible for inclusion, of whom 124 had curative operations for colon cancer and 68 had curative operations for rectal cancer. Figure 1 demonstrates the study flow chart.

Baseline patient characteristics

Patients With Colon Cancer

The mean age of the patients with colon cancer was 67.44 (SD: 1.07). In terms of gender, 67 out of 124 (54%) were male, and 57 out of 124 (46%) were female. In terms of ASA status, 8 out of 124 (7%) were classed as ASA 1, 62 out of 124 (50%) as ASA 2, 51 out of 124 (41%) as ASA 3, and 3 out of 124 (2%) as ASA 4. In terms of TNM stage, 26 out of 124 (21%) patients had stage I disease, 43 out of 124 (35%) had stage II disease, and 55 out of 124 (44%) had stage III disease. The histological assessment showed well-differentiated adenocarcinoma in 3 out of 124 (2%) patients, moderately differentiated adenocarcinoma in 109 out of 124 (88%) patients, poorly differentiated adenocarcinoma in 7 out of 124 (6%) patients, and other pathologies in 5 out of 124 (4%) patients. In terms of setting, 117 out of 124 (94%) patients underwent elective operations, and 7 out of 124 (6%) patients underwent emergency operations. R0 resection was achieved in 122 out of 124 (98%) patients, and the mean number of harvested lymph nodes was 20.22 (SD: 0.81), with the achievement of >12 harvested lymph nodes in 114 out of 124 (92%) patients. The mean number of positive lymph nodes was 1.42 (SD: 0.28). The mean tumour size and specimen lengths were 39.84 mm (SD: 1.48) and 254.1 mm (SD: 17.3), respectively. The length of the specimen was >120 mm in 114 out of 124 (92%) patients.

Patients With Rectal Cancer

The mean age of the patients with rectal cancer was 68.16 (SD: 1.14). In terms of gender, 40 out of 68 (59%) were male, and 28 out of 68 (41%) were female. In terms of ASA status, 5 out of 68 (7%) were classed as ASA 1, 45 out of 68 (66%) as ASA 2, 18 out of 68 (27%) as ASA 3, and 0 out of 68 (0%) as ASA 4. In terms of TNM stage, 18 out of 68 (27%) patients had stage I disease, 13 out of 68 (19%) had stage II disease, and 37 out of 68 (54%) had stage III disease. The histological assessment showed well-differentiated adenocarcinoma in 2 out of 68 (3%) patients, moderately differentiated adenocarcinoma in 61 out of 68 (90%) patients, poorly differentiated adenocarcinoma in 4 out of 68 (6%) patients, and other pathologies in 1 out of 68 (1%) patients. In terms of setting, 67 out of 68 (99%) patients underwent elective operations, and 1 out of 68 (1%) patients underwent an emergency operation. R0 resection was achieved in 65 out of 68 (96%) patients, and the mean number of harvested lymph nodes was 19.96 (SD: 1.46), with the achievement of >12 harvested lymph nodes in 54 out of 68 (79%) patients. The mean number of positive lymph nodes was 2.19 (SD: 1.07). The mean tumour size and specimen lengths were 32.97 mm (SD: 1.54) and 193.9 mm (SD: 16.6), respectively. The length of the specimen was >120 mm in 54 out of 68 (79%) patients (Table 1).

**Table 1:** Baseline characteristics of the included patients. ASA: American Society of Anaesthesiologists; TNM: Tumour, Nodal status, Metastasis.

	Patients with colon cancer	Patients with rectal cancer
Number of patients	124	68
Mean age (SD)*	67.44 (1.07)	68.16 (1.14)
Male	67 out of 124	40 out of 68
Female	57 out of 124	28 out of 68
ASA		
1	8 out of 124	5 out of 68
2	62 out of 124	45 out of 68
3	51 out of 124	18 out of 68
4	3 out of 124	0 out of 68
5	0 out of 124	0 out of 68
TNM Stage		
I	26 out of 124	18 out of 68
II	43 out of 124	13 out of 68
III	55 out of 124	37 out of 68
Histology		
Well-differentiated adenocarcinoma	3 out of 124	2 out of 68
Moderately differentiated adenocarcinoma	109 out of 124	61 out of 68
Poorly differentiated adenocarcinoma	7 out of 124	4 out of 68
Other	5 out of 124	1 out of 68
Setting		
Elective	117 out of 124	67 out of 68
Emergency	7 out of 124	1 out of 68
R0 resection	122 out of 124	65 out of 68
Mean number of harvested lymph nodes (SD)*	20.22 (0.81)	19.96 (1.46)
>12 harvested lymph nodes	114 out of 124	54 out of 68
Mean number of positive lymph nodes (SD)*	1.42 (0.28)	2.19 (1.07)
Mean tumour size in mm (SD)*	39.84 (1.48)	32.97 (1.54)
Mean specimen length in mm (SD)*	254.1 (17.3)	193.9 (16.6)
Specimen length > 120 mm	114 out of 124	54 out of 68

Regression analyses

Patients With Colon Cancer

Regression analysis showed that in patients with colon cancer, tumour size was the independent predictor of the number of harvested lymph nodes (coefficient: 0.2275, 95% CI: 0.1361-0.3190, P<0.001), the number of positive lymph nodes (coefficient: 0.0568, 95% CI: 0.0235-0.0902, P=0.001), and lymphocytic infiltration (OR: 1.0414, 95% CI: 1.0100-1.0738, P=0.009). However, it did not predict R0 resection (OR: 1.0687, 95% CI: 0.8535-1.3382, P=0.563), specimen length (coefficient: 1.74, 95% CI: -0.40-3.88, P=0.111), specimen length >120 mm (OR: 1.0396, 95% CI: 0.9814-1.1012, P=0.186), >12 harvested lymph nodes (OR: 1.0420, 95% CI: 0.9860-1.1012, P=0.145), venous invasion (OR: 1.0192, 95% CI: 0.9962-1.0428, P=0.103), and five-year overall survival (OR: 1.0145, 95% CI: 0.9685-1.0628, P=0.543).

Patients With Rectal Cancer

Regression analysis showed that in patients with rectal cancer, tumour size was the independent predictor of the number of harvested lymph nodes (coefficient: 0.429, 95% CI: 0.203-0.654, P<0.001), and the number of positive lymph nodes (coefficient: 0.3163, 95% CI: 0.1459-0.4867, P<0.001). However, it did not predict R0 resection (OR: 1.2968, 95% CI: 0.9447-1.7802, P=0.108), specimen length (coefficient: 0.223, 95% CI: -1.323-1.769, P=0.774), specimen length >120 mm (OR: 1.0232, 95% CI: 0.9693-1.0802, P=0.405), >12 harvested lymph nodes (OR: 1.0595, 95% CI: 0.9956-1.1276, P=00.069), lymphocytic infiltration (OR: 0.9934, 95% CI: 0.8827-1.1180, P=0.912), venous invasion (OR: 1.0384, 95% CI: 0.9922-1.0868, P=0.105), and five-year overall survival (OR: 0.9745, 95% CI: 0.9162, 1.0366, P=0.413) (Table 2).

**Table 2:** Results of linear and binary logistic regression analyses. $ results of binary logistic regression analysis is presented as OR and results of linear regression analysis is presented as coefficient.

Dependent variable	Independent variable (tumour size)			
	Patients with colon cancer (N = 124)		Patients with rectal cancer (N = 68)	
	OR or Coefficient^$^	P-value	OR or Coefficient^$^	P-value
R0 resection	OR: 1.0687 (0.8535, 1.3382)	0.563	OR: 1.2968 (0.9447, 1.7802)	0.108
Specimen length	Coefficient: 1.74 (-0.40, 3.88)	0.111	Coefficient: 0.223 (-1.323, 1.769)	0.774
Specimen length >120mm	OR: 1.0396 (0.9814, 1.1012)	0.186	OR: 1.0232 (0.9693, 1.0802)	0.405
Number of harvested lymph nodes	Coefficient: 0.2275 (0.1361, 0.3190)	<0.001	Coefficient: 0.429 (0.203, 0.654)	<0.001
>12 harvested lymph nodes	OR: 1.0420 (0.9860, 1.1012)	0.145	OR: 1.0595 (0.9956, 1.1276)	0.069
Number of positive lymph nodes	Coefficient: 0.0568 (0.0235, 0.0902)	0.001	Coefficient: 0.3163 (0.1459, 0.4867)	<0.001
Lymphocytic infiltration	OR: 1.0414 (1.0100, 1.0738)	0.009	OR: 0.9934 (0.8827, 1.1180)	0.912
Venous invasion	OR: 1.0192 (0.9962, 1.0428)	0.103	OR: 1.0384 (0.9922, 1.0868)	0.105
One-year overall survival	OR: 0.9392 (0.8927, 0.9880)	0.015	OR: 1.0965 (0.9614, 1.2507)	0.17
Three-year overall survival	OR: 0.9789 (0.9451, 1.0139)	0.234	OR: 0.9935 (0.9179, 1.0752)	0.871
Five-year overall survival	OR: 1.0145 (0.9685, 1.0628)	0.543	OR: 0.9745 (0.9162, 1.0366)	0.413

**Figure 2:**
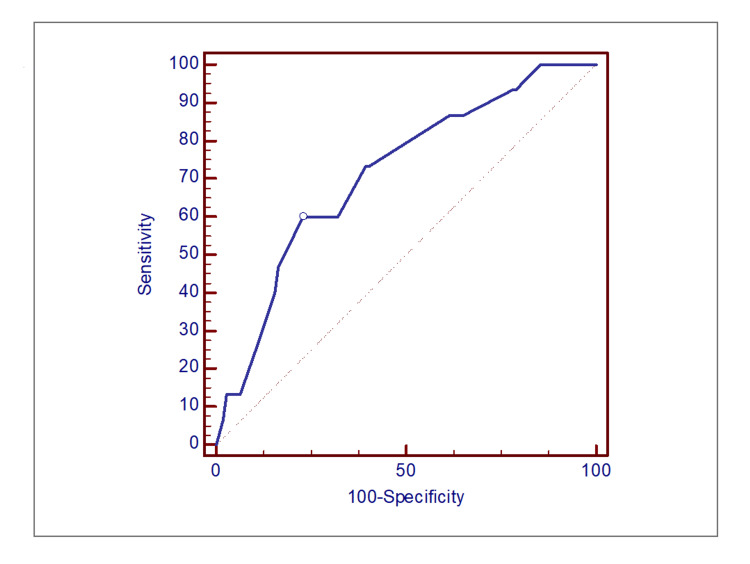
Results of ROC curve analysis for tumour size as a predictor of lymphocytic infiltration in patients with colon cancer. ROC: Receiver operating characteristic.

ROC curve analysis seen in Figure 2 identified that in patients with colon cancer, a tumour size of 48 mm was the cut-off value for lymphocytic infiltration. The colonic tumour size had AUC of 0.71 (95% CI: 0.63-0.79, P=0.0022) with sensitivity of 60% (95% CI: 32%-84%) and specificity of 77% (95% CI: 68%-85%).

## Discussion

The preferred management of non-metastatic colon cancer is excision of the tumour and regional draining lymph nodes. Post-surgical treatment is closely related to the TNM staging system of the AJCC and Union for International Cancer Control for CRC [4], and differentiation of the tumour and lymphovascular invasion [10].

Depth of the tumour penetration (T), regional lymph nodes involvement (N) and distant metastasis (M) are major parameters predicting the prognosis of CRC patients. However, the literature shows that tumour staging may be more accurate; the prognosis may be more predictable as the number of harvested lymph nodes increases [11-13].

Several studies show that the vertical spread of the tumour on the bowel wall is related to the number of positive lymph nodes and a poorer prognosis [14-16]. However, the relationship between horizontal tumour diameter and prognosis is still controversial [14-16]. Furthermore, few studies in gastric and colonic cancer indicate that the horizontal extension of the tumour could be an important prognostic factor [3,17,18].

We conducted a retrospective analysis of prospectively collected data on a cohort of patients operated by a single colorectal surgeon to evaluate the predictive significance of tumour size in patients undergoing curative surgery for CRC.

Analysis of 192 patients (124 with colon cancer and 68 with rectal cancer) showed that in patients with colon cancer, tumour size was an independent predictor of the number of harvested lymph nodes, the number of positive lymph nodes, and lymphocytic infiltration. However, it did not predict R0 resection, specimen length, specimen length >120 mm, >12 harvested lymph nodes, venous invasion, and five-year overall survival. In patients with rectal cancer, tumour size was an independent predictor of the number of harvested lymph nodes and the number of positive lymph nodes. However, it did not predict R0 resection, specimen length, specimen length >120 mm, >12 harvested lymph nodes, lymphocytic infiltration, venous invasion, and five-year overall survival.

The results of the current study suggest that tumour size on its own may not have a significant predictive value in oncological or survival outcomes in patients undergoing curative surgery for colon or rectum cancer. Our results are consistent with the findings of the studies by Miller W et al. and Crozier JE et al. [5,6]. In fact, Miller W et al. conducted a retrospective analysis of 391 patients with primary colon cancer and concluded that colon carcinoma metastasis and survival are independent of tumour size [5]. Consistent with this, Crozier JE et al. investigated tumour diameter and pathological characteristics of resected specimens of 227 patients who underwent curative operations for CRC and concluded that tumour size does not predict survival in patients undergoing curative operations for CRC [6].

Unlike the current study's findings, some authors argued that colorectal tumour size has prognostic significance [3,7,8,19]. For example, Saha S et al. analysed 300,386 patients with colon cancer and concluded that colonic tumour size is associated with negative long-term survival [7]. In another study, Kornprat P et al. evaluated 381 CRC specimens and identified tumour size as an independent prognostic parameter in patients with CRC [3]. Consistent with these two studies, Dai W et al. investigated 4057 patients with CRC and concluded that tumour size could independently predict survival in patients with CRC [19].

Although some studies have investigated the association between tumour size and survival outcomes, the relationships between the tumour size and other parameters investigated in our study, such as R0 resection, specimen length, specimen length >120 mm, number of harvested lymph nodes, >12 harvested lymph nodes, number of positive lymph nodes, lymphocytic infiltration, and venous invasion have not been assessed previously. Our results showed that tumour size could predict the number of harvested lymph nodes and positive lymph nodes; nevertheless, it did not affect the achievement of >12 harvested lymph nodes. Moreover, although our results suggested that colonic tumour size may predict lymphocytic infiltration in patients with colon cancer, this did not translate into a prediction of five-year overall survival. Therefore, the clinical significance of these findings remains debatable.

The lymph node involvement is used to determine adjuvant therapy in CRC patients. The relationship between the size of the tumour and lymph node invasion could be important. According to our data, there was a statistically significant relationship between the tumour size and the number of positive lymph nodes, coefficient: 0.568 (0.0235, 0.0902), P-value 0.001, in the colon and rectal cancers and coefficient: 0.3163 (0.1459, 0.4867) and p-value < 0.001 on linear regression analysis. Although some studies in the literature support our data [3,18,20], other studies concluded that tumour size is not an important factor in determining lymph node involvement [6,21].

The other significant finding in our study was that tumour size could predict the number of harvested lymph nodes in colon cancer. Colon cancer, coefficient: 0.2275 (0.1361, 0.3190) and p-value <0.001 and rectal cancer, coefficient: 0.429 (0.203, 0.654) and p-value <0.001. There is no major evidence in the literature to support this finding. It is a new finding, and the number of harvested nodes improves the quality of TNM staging and decision for adjuvant chemotherapy. As this is a new finding, we recommend investigating this in future studies.

The third important finding in our study is whether tumour size can predict lymphocytic infiltration. This was significant in colon cancer only OR: 1.0414 (1.0100, 1.0738), p-value 0.009. However, no significant relationship was found in the rectal cancer group. As we know that lymphovascular invasion is a poor prognostic factor, we could not find evidence that tumour size can predict lymphocytic infiltration.

More recent emerging approaches, including the use of molecular and genetic markers, may eventually provide prognostic and predictive information. For example, recent studies in CRC biology have been using potential molecular and genetic markers to stratify early-stage CRC patients. Microsatellite instability (MSI) genetic signature is an example which is found to be associated with a better prognosis in these patients. However, the proper validation of immunohistochemistry or gene expression analysis in clinical trials is lacking [22].

This study is associated with some limitations. First, the retrospective nature of the current study would subject our results to the inevitable risk of selection bias. This study was performed in a single centre, and a single colorectal surgeon performed all the operations. Although this would control the confounding effect related to the operating surgeon and the surgical setting, it may limit the generalisability of our findings. Second, considering the relatively small sample size of this study, the likelihood of type 2 error cannot be excluded. Third, the five-year survival data were unavailable for a significant proportion of patients in both colon and rectal cancer groups. Although it does not affect our conclusions regarding associations between the tumour size and most of the oncological outcomes, it would definitely subject our conclusion regarding five-year survival to potential type 2 error.

## Conclusions

The results of the current study suggest that tumour size on its own may not have a significant predictive value in oncological or survival outcomes in patients undergoing curative surgery for colon or rectum cancer. However, our study sheds light on two new observations related to tumour size. In our sample, tumour size could predict the number of harvested lymph nodes in colon cancer. In addition, it could significantly predict lymphocytic infiltration in colon cancer only. Further studies with a larger number of patients and adequate long-term follow-up are warranted for further validation of the effect of tumour size on oncologic outcomes. It is hoped that new modalities with better prognostic accuracy for CRC will emerge to help improve the decision-making and tailoring of surgeries to each patient's needs and circumstances.
